# Facioscapulohumeral muscular dystrophy’s game of homeodomains: therapy wants a biomarker as a sword wants a whetstone

**DOI:** 10.1093/braincomms/fcad235

**Published:** 2023-09-02

**Authors:** Michael Kyba, Darko Bosnakovski

**Affiliations:** Lillehei Heart Institute, University of Minnesota, Minnesota 55455, USA; Department of Pediatrics, University of Minnesota, Minnesota 55455, USA; Lillehei Heart Institute, University of Minnesota, Minnesota 55455, USA; Department of Pediatrics, University of Minnesota, Minnesota 55455, USA

## Abstract

This scientific commentary refers to ‘The FSHD muscle-blood biomarker: a circulating transcriptomic biomarker for clinical severity in facioscapulohumeral muscular dystrophy’, by Banerji *et al*. (https://doi.org/10.1093/braincomms/fcad221).

**This scientific commentary refers to ‘The FSHD muscle-blood biomarker: a circulating transcriptomic biomarker for clinical severity in facioscapulohumeral muscular dystrophy’, by Banerji *et al*. (https://doi.org/10.1093/braincomms/fcad221).**

Although facioscapulohumeral muscular dystrophy (FSHD) is one of the most common muscular dystrophies, and is understood quite well at the molecular genetic level, it is still very enigmatic at the cellular and tissue levels. The current consensus is that leaky expression of the normally silent *DUX4* gene somehow provokes irreversible muscle alterations characterized by immune cell infiltration, wasting, fibro-adipose matrix deposition and muscle weakness.^1^ The underling genetics of the disease leads to impairment of repeat-induced silencing of the D4Z4 repeat, typically due to deletion of repeats on a permissive chromosome 4, i.e. one bearing a poly A signal immediately downstream of the last D4Z4 repeat.^2^ In ∼5% of the cases, classified as FSHD2, the de-repression occurs primarily due to second site mutations in epigenetic modifiers, most commonly *SMCHD1*.^3^ Clinical manifestation of FSHD is highly heterogeneous regarding age of onset, penetrance, muscle group affected, progression and severity which ranges from non-ambulatory to asymptomatic carriers.^4^ While as of this writing, there are no specific and effective therapies approved for FSHD, the field is burgeoning with DUX4-targeted approaches aiming to reduce DUX4 RNA or protein levels, or to inhibit the DUX4 protein function, with clinical trials already completed, ongoing or expected to begin enrolling soon.

While the genetics predicts that DUX4 expression causes FSHD, it has not proven possible to convincingly visualize DUX4 protein *in situ*. Thus, we cannot be entirely confident which cell types are actually expressing DUX4 in affected individuals. Although myoblast cultures show evidence of DUX4 expression in rare nuclei,^5^ they do not rule out similar leaky expression in other cell types, such expression being not unexpected given that the epigenetic changes that affect the D4Z4 locus occur in all cells; indeed, epigenetic studies on blood cells of FSHD patients were used extensively to elucidate the molecular mechanism of the disease.^6^ The possibilities of DUX4 expression in non-myogenic cells of muscle, in cells of an organ other than muscle, or even past expression leading to cytopathological alterations that persist in the absence of continued DUX4 expression^7^ are all technically live options. Thus, for the therapies in development and testing, evaluation of DUX4 protein expression *in situ* will not be a useful early marker of appropriate target engagement.

The natural history of FSHD presents a further major difficulty in therapy development: the disease is very slowly progressing in most individuals, meaning that for interventions that halt disease progression (as opposed to reversing it), large sample sizes and long times are necessary to have any chance at revealing efficacy, even in early phase studies. This is assuming that physiological and/or functional outcomes guide such studies. For efficacy-based triage of the most promising therapies in a rare disease such as FSHD, surrogate markers of efficacy, so-called biomarkers of disease, can be very valuable. Reduction in a marker that is strongly correlated with disease progression predicts a reduced rate of disease progression prior to the physiological changes that actually define disease progression.

Dozens of studies identify serum proteins, circulating mRNA, complement components and transcriptomes as potential FSHD biomarkers.^1^ However, practical applicability for the majority of markers is uncertain due to the lack of reproducibility. The blame for the inconsistent results may be due to limited cohorts, a wide degree of heterogeneity in disease presentation, and most often to the origin of the biopsy material. Because the DUX4 protein cannot be detected directly *in situ*, a set of the most-responsive DUX4 target genes might be thought of as a good surrogate for its presence, and indeed, DUX4 target genes do represent the currently most-favoured biomarkers. However, because of the extremely low or rare expression of DUX4, these target genes are not expressed robustly, and biomarker sets based on DUX4 target genes cannot unequivocally discriminate all patients from controls, even in the best case, where biopsies are taken from regions of likely oedema indicated by MRI STIR+, known to have the best expression of DUX4 target genes.^8^

While a set of genes based on the DUX4 transcriptome has the advantage of being directly related to the target of the therapy, the group of Banerji and Zammit have taken a different approach, based on the target genes of a different homeodomain transcription factor: PAX7. One of the earliest studies on DUX4 pointed out that the homeodomains of DUX4 and PAX7 are highly related in sequence and that DUX4 demonstrated an interesting genetic interaction with PAX7, namely that PAX7 strongly interfered with the cytotoxicity of DUX4.^9^ The mechanism of this interaction was originally thought to be competition for binding to the same DNA sequences, however, commonly-bound targets have not been discovered, rendering this competition mechanistically unexplained to date. Nevertheless, Banerji and Zammit evaluated PAX7 target genes in FSHD biopsies and found an inverse correlation: genes up and downregulated by PAX7 tended to be down and upregulated, respectively, in FSHD biopsies.^10^ In this issue of *Brain Communications*, they mine this PAX7 gene set more deeply, in an attempt to explore whether a subset of these genes might serve as a useful biomarker for disease severity or progression in FSHD.^11^

To begin, they set up a direct competition between the target gene sets of the two homeodomain proteins, PAX7 and DUX4, as well as an unrelated gene set based on genes differentially expressed in EBV-transformed B-cell lines from FSHD and control patients; in which a single score output variable was computed for each, and the correlation of that variable generated from RNA-seq from muscle biopsies of FSHD and control participants, including paired biopsies from the FSHD group on inflamed (based on MRI, called TRIM+) and non-inflamed muscles (TIMR−), was tested on discriminating presence and degree of disease as well as overall clinical severity. On the DUX4 side were actually three somewhat overlapping gene sets, based on three different studies in which DUX4 target genes were identified. Out of this five-player battle-royale emerged one clear winner: the PAX7 score. Remarkably, the PAX7 score was able to discriminate between not just FSHD versus control but also between inflamed versus non-inflamed muscle, and correlated with disease severity measured by the Ricci and Lamperti assessment tools.

They also obtained RNA from peripheral blood mononucleocytes from 14 FSHD and 14 control donors, and although none of the five gene sets discriminated FSDH from control blood (14 + 14), the PAX7 score showed a correlation between TIRM− muscle and blood, therefore, they refined the 601 genes that comprise the PAX7 score (311 up and 290 down), to a smaller set of 143 genes (64 up and 79 down) based on individual correlations to disease severity in the donors. This refined gene set now correlated the score from blood with both disease state and disease severity and was also able to distinguish TRIM+ from TIRM− FSHD muscle biopsies. The authors therefore named this gene set or more specifically the value calculated from this gene set, the ‘muscle-blood biomarker’.

They then tested this muscle-blood biomarker on independently generated RNA-seq data from primary tissue samples. Remarkably, it was able to discriminate between year 1 and year 2 samples from the Wong longitudinal muscle biopsy transcriptomic dataset,^12^ meaning that it might be used to measure disease progression, and potentially mitigation thereof in clinical trials. Key, however, it was also successful on independent blood RNA-seq data, discriminating mild versus severe in the Signorelli study of blood transcriptional profiles of FSHD patients,^13^ particularly in the older patients of this study. They thus present this 143 gene-based ‘muscle-blood biomarker’ as a potentially useful minimally invasive tool in assessment of disease progression in future therapeutic studies (as well as in the recent Losmapimod study, whose RNA-seq data has still not been made public). Such a tool would be extremely valuable for predicting clinical efficacy.

So does the PAX7 sword really cut the sharpest? A few considerations might give the DUX4 side some hope. The first would be that in a clinical study that attempts to inhibit DUX4, it will still clearly be necessary to evaluate DUX4 target genes, if only to demonstrate that the therapy is acting on target. In fact, since we do not understand how the inverse-PAX7 pattern comes about (neither blood cells nor differentiated myofibres express PAX7), its predictive value in terms of disease progression in the presence of therapies that target DUX4 is impossible to speculate on. As clinical studies proceed, this will become clear (providing sponsors publish their RNA-seq data). Finally, the authors did not attempt to refine the DUX4 target genes to a smaller set with potentially greater discriminatory power, thus, it remains possible that a DUX4 muscle-blood biomarker might yet emerge. The approach of correlating a single variable from a set of genes to disease state is somewhat unconventional, and indeed, when the authors used the Globaltest algorithm, which computes association over a set without compressing into a single variable, they found that the Choi DUX4 gene set^10,14^ did have the power to discriminate between TRIM+ versus TRIM-neg isogenic biopsies. Thus, the game is not quite over, but the PAX7 target gene set is clearly a force to be reckoned with.
